# Unraveling the Multifaceted Role of the miR-17-92 Cluster in Colorectal Cancer: From Mechanisms to Biomarker Potential

**DOI:** 10.3390/cimb46030120

**Published:** 2024-02-28

**Authors:** Hakeemah H. Al-nakhle

**Affiliations:** Department of Clinical Laboratory Sciences, College of Applied Medical Sciences, Taibah University, Al-Madinah Al-Monawarah 42353, Saudi Arabia; hnakhly@taibahu.edu.sa

**Keywords:** colorectal cancer, miR-17-92 cluster, diagnostic marker, prognostic marker, gene regulation, signaling pathway

## Abstract

Colorectal cancer (CRC) is a complex disease driven by intricate mechanisms, making it challenging to understand and manage. The miR-17-92 cluster has gained significant attention in CRC research due to its diverse functions and crucial role in various aspects of the disease. This cluster, consisting of multiple individual miRNAs, influences critical processes like tumor initiation, angiogenesis, metastasis, and the epithelial–mesenchymal transition (EMT). Beyond its roles in tumorigenesis and progression, miR-17-92′s dysregulation in CRC has substantial implications for diagnosis, prognosis, and treatment, including chemotherapy responsiveness. It also shows promise as a diagnostic and prognostic biomarker, offering insights into treatment responses and disease progression. This review provides a comprehensive overview of recent advancements and the context-dependent role of the miR-17-92 cluster in colorectal cancer, drawing from the latest high-quality published data. It summarizes the established mechanisms governing miR-17-92 expression and the molecular pathways under its influence. Furthermore, it examines instances where it functions as an oncogene or a tumor suppressor, elucidating how cellular contexts dictate its biological effects. Ultimately, miR-17-92 holds promise as a biomarker for prognosis and therapy response, as well as a potential target for cancer prevention and therapeutic interventions. In essence, this review underscores the multifaceted nature of miR-17-92 in CRC research, offering promising avenues for enhancing the management of CRC patients.

## 1. Introduction

Colorectal cancer (CRC) is a significant global health concern, ranking as the third leading cause of cancer-related deaths, with an alarming projected increase in cases by 2040 [1,2]. Its pathogenesis involves complex interactions between environmental and genetic factors [3,4]. To address the challenge of early detection and intervention, microRNAs (miRNAs) have emerged as potential biomarkers of great importance.

miRNAs are small non-coding RNA molecules that regulate gene expression by binding to target mRNA molecules [3]. Dysregulation of the miRNA biogenesis machinery has been identified in cancer cells, influencing tumor development and progression [4]. miRNAs exhibit diverse roles, acting as both oncogenes and tumor suppressors, depending on the context [4]. Among these, the miR-17-92 cluster has gained attention for its role in CRC.

The miR-17-92 cluster is upregulated in colon cancer and contributes to tumor development by promoting angiogenesis through the suppression of genes like thrombospondin 1 (TSP 1) and connective tissue growth factor (CTGF) [5]. Specific miRNAs within the cluster, such as miR-18a and miR-20a, are associated with an unfavorable prognosis in CRC [6]. miR-92 has been linked to reduced apoptosis in CRC patients [7]. In addition, the increased expression of miR-17 is associated with decreased survival in CRC patients [8]. Elevated miR-17 and miR-92 levels have been observed in both tumor tissues and serum samples from CRC patients, distinguishing them from healthy controls and patients with other conditions [9]. Furthermore, miR-18a expression correlates with adenomatous polyposis coli (APC) mutations in CRC samples [10].

This comprehensive literature review aims to elucidate the multifaceted roles of the miR-17-92 cluster in CRC biology. By consolidating existing knowledge, it seeks to provide insights into the molecular mechanisms underlying its influence on CRC development, progression, and treatment response. Additionally, this review evaluates the clinical significance of the miR-17-92 cluster as a diagnostic biomarker and potential therapeutic target in CRC patients.

## 2. miRNA Biology and Biogenesis

**Figure 1:**
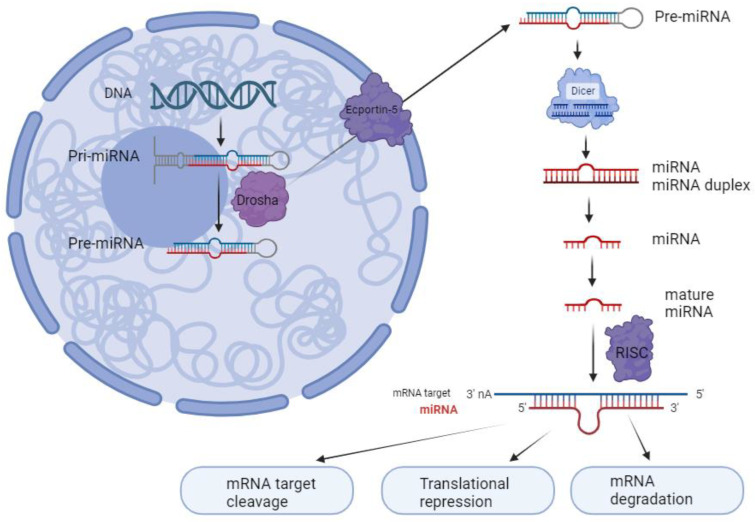
miRNA biosynthesis process. miRNA is generated by RNA polymerase II as pri-miRNA. These pri-miRNAs are then cleaved into pre-miRNAs by a complex of microprocessors that includes DROSHA. Subsequently, pre-miRNAs are transported from the nucleus to the cytoplasm via exportin-5, where they undergo further processing by Dicer to form double-stranded miRNA. Once double-stranded, the miRNA binds to the argonaute protein within the RNA-induced silencing complex (RISC), where it becomes separated into its mature strand. This mature strand remains within the RISC and is responsible for binding to target mRNA molecules to regulate gene expression, while the other passenger strand is enzymatically hydrolyzed (created with BioRender.com, accessed on 5 January 2024).

miRNAs are small non-coding RNA molecules with ∼22 nucleotides [11]. They can regulate gene expression by binding to the 3′ untranslated regions (3′ UTRs) of the mRNAs on target molecules [3]. As shown in Figure 1, in general, the miRNA biogenesis starts with transcribing a long primary miRNA (pri-miRNA) by RNA polymerase II. This pri- miRNA is then cleaved into a precursor miRNA (pre-miRNA) within the nucleus of the cell by endonucleases such as DROSHA [12]. Then, exportin-5 (XPO5) transports the pre-miRNA to the cytoplasm, where it is further cleaved into an miRNA duplex by a ribonuclease, DICER1. Once in the cytoplasm, this RNA duplex forms a ribo-protein complex with the argonaute (AGO) protein, leading to the generation of the RNA-induced silencing complex (RISC). From this duplex, one strand, known as mature miRNA, accumulates preferentially, while the other is degraded as a passenger strand [12]. Usually, miRNAs inhibit gene expression by interacting with miRNA response elements (MREs). These MREs are usually located in the target mRNAs’ 3′ UTRs. It has been reported that the MREs can be found in the 5′ UTR or open reading frame [13]. Mutations that change the machinery for processing miRNAs have been identified in cancer cells [4]. These mutations lead to the disruption of the regulation of miRNAs, influencing the development and progression of tumors. Specific components of the miRNA biogenesis machinery, such as XPO5, DICER1, and TARBP2, have been identified as tumor suppressor genes when their function is reduced. Moreover, primary colon cancer tumors with microsatellite instability have been found to contain mutations in XPO5 and TARBP2 that impair miRNA processing and enhance cellular transformation [14].

miRNAs play important roles in the initiation and progression of various cancers including lung, colorectal, breast, and pancreatic [4]. A single miRNA can control signaling pathways associated with tumors by targeting hundreds of mRNAs. Mutations that disrupt the miRNA processing machinery have been observed in cancer cells, leading to dysregulation of miRNAs that may contribute to tumor development and progression [4]. miRNAs exhibit diverse roles in cancers; some act as oncogenes while functioning as tumor suppressors in others. For example, miR-92 acts as an oncogene by inhibiting the tumor suppressor gene, ERβ1, in breast cancer [15], whereas other miRNAs have also been found to promote breast cancer invasion [16]. Moreover, miR-125b is oncogenic in malignant hematopoietic cells but exhibits tumor-suppressive effects in many solid tumors [17]. The miR-17-92 cluster also exerts opposing effects on tumors, targeting various mRNA molecules involved in different pathways.

## 3. Structure and Organization of the miR-17-92 Cluster Families

**Figure 2:**
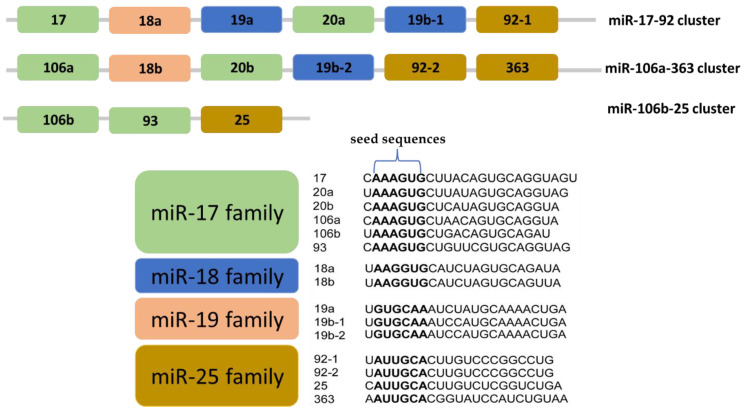
The components of the miR-17-92 cluster and its two paralogous clusters. The mature miRNAs within these three clusters can be categorized into four distinct miRNA families: miR-17, miR-18, miR-19, and miR-92 families, determined by their seed sequences.

The polycistronic miR-17-92 cluster, located on chromosome 13q31.3, is frequently amplified in various cancer types, including lung cancer, CRC, and B-cell lymphomas [18]. This cluster is transcribed as a single primary transcript, generating six distinct mature miRNAs: miR-17, miR-18a, miR-19a, miR-19b, miR-20a, and miR-92a. Notably, it possesses two paralogs known as the miR-106b-25 and miR-106a-363 clusters, adding complexity to its functional repertoire [19] (Figure 2).

The miR-106b-25 cluster, situated on chromosome 7q22.1, encodes miR-25, miR-93, and miR-106b. The elevated expression of this cluster has been linked to cell cycle arrest, apoptosis, and the activation of tumor-initiating cells (TICs) in breast cancer [20]. Furthermore, these microRNAs are overexpressed in various cancers, such as prostate carcinoma, esophageal cancer, gastric carcinoma, and hepatocellular carcinoma (HCC), underscoring their potential as oncogenes [21].

On the other hand, the miR-106a-363 cluster resides on chromosome Xq26.2 and encompasses miR-106a, miR-18b, miR-363, miR-20b, miR-19b-2, and miR-92a-2. This particular cluster plays a role in cancer development; for example, miRNA20b promotes cell growth in breast cancer [22], while its expression is reduced in colorectal tumors [23]. Additionally, head and neck cancers exhibit decreased expression of miR-363-5p [24]. Conversely, the overexpression of miR363-5p inhibits cell proliferation in HCC by targeting S1PR1 [25].

Although the miRNAs within these three paralogous clusters exhibit high sequence similarity, their functions notably intersect. Specifically, the individual knockout of either the miR-106b-25 or miR-106a-363 clusters in mice does not result in observable phenotype changes [26]. In contrast, mice lacking the miR-17-92 cluster exhibit defects in their heart and/or lungs, leading to lethality [26]. The simultaneous knockout of both the miR-17-92 and miR-106b-25 clusters, or all three clusters, results in more severe mid-gestation defects and lethality [26]. These differing phenotypes upon cluster knockout suggest functional redundancy among the paralogs of the miR-17-92 family.

## 4. Mechanisms Underlying Altered miR-17-92 Expression in Colorectal Cancer

One of the mechanisms contributing to the overexpression of the miR-17-92 cluster in CRC is the genetic amplification of its genomic locus. This amplification is associated with CRC progression and plays a crucial role in the transition from colorectal adenoma to carcinoma. It is also implicated in the promotion of 13q chromosomal gain [27]. Additionally, during the transition from colorectal adenoma to adenocarcinoma, there is a notable increase in miR-17-92 cluster expression, which is linked to DNA copy number gains at the miR17-92 locus on chromosome 13q31 and heightened c-Myc expression. Among the six members of the miR-17-92 cluster, all except miR-18a show significantly higher expression in colorectal tumors with miR-17-92 locus amplification compared to tumors lacking this amplification. Unsupervised cluster analysis can differentiate tumors based on miR-17-92 locus gain, and a significant correlation exists between c-Myc expression and the expression of these six miRNAs [18].

Another mechanism leading to the increased expression of miR-17-92 in colorectal cancer involves the activation of oncogenic signaling pathways, particularly the Wnt/β-catenin pathway. miR-92a has been identified as a key player in promoting Wnt/β-catenin signaling activity by directly targeting KLF4, GSK3β, and DKK3, all of which are negative regulators of the Wnt/β-catenin signaling cascade. Furthermore, the study suggests that the IL-6/STAT3 pathway enhances miR-92a expression by directly targeting its promoter, resulting in the activation of Wnt/β-catenin signaling and the subsequent promotion of stem-like traits in colorectal cancer cells. These findings underscore the critical role of the IL-6/STAT3/miR-92a/Wnt/β-catenin pathway in regulating the stem-cell-like characteristics of colorectal cancer cells and offer a potential target for colorectal cancer therapy [28].

It is important to emphasize that the regulation of miR-17-92 in CRC is multifaceted, and multiple factors may collaborate to modulate its expression. A comprehensive understanding of the specific mechanisms involved in individual cases of CRC can provide valuable insights into the disease and identify potential therapeutic targets.

## 5. Involvement of the miR-17-92 Cluster in Signaling Pathways in Colorectal Cancer Pathogenesis

**Figure 3:**
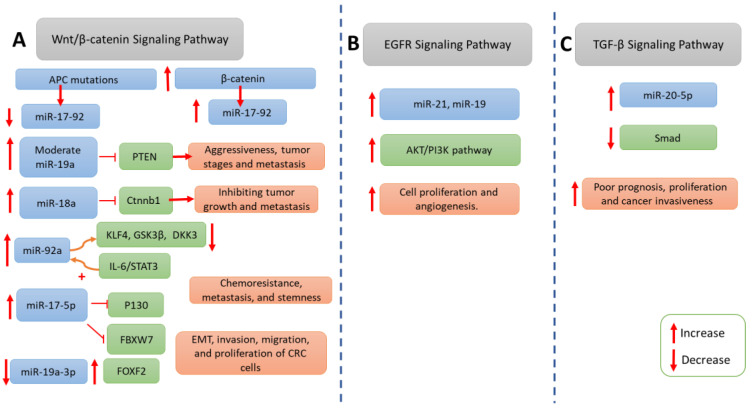
The role of the miR-17-92 cluster in signaling pathways during the progression of CRC. (**A**) The Wnt/β-catenin signaling pathway in colorectal carcinogenesis. The miR-17-92 cluster is significantly affected by the APC/β-catenin pathway, being downregulated by APC mutations and upregulated upon enforced β-catenin expression. Moreover, activated β-catenin directly promotes miR-17-92 expression. Alterations in miR-17-92 levels, particularly miR-19a, correlate with CRC aggressiveness, tumor stages, and β-catenin levels. Furthermore, the miR-17-92 cluster’s multifaceted role in CRC involves differential effects based on expression levels, influencing tumor metastasis and growth. Additionally, miR-92a, miR-17-5p, miR-19a-3p, and miR-20a contribute to CRC progression by affecting their targets through various mechanisms, including chemoresistance, metastasis, and stemness. (**B**) The EGFR pathway plays a crucial role in cellular physiology, activating signaling pathways like PI3K/AKT upon activation. miR-21 and miR-19 modulate the AKT–PI3K axis, affecting cell proliferation and angiogenesis. (**C**) The TGF-β/Smad pathway. Disruption of Smad-4 correlates with poor prognosis, with miRNA-20-5p notably silencing Smad, promoting proliferation, and increasing CRC invasiveness.

Numerous proteins pivotal to critical signaling pathways associated with CRC, including the Wnt/β-catenin, EGFR, and TGF-b signaling pathways, the phosphatidylinositol-3-kinase (PI-3-K) pathways, KRAS, p53, extracellular matrix regulators, and EMT transcription factors, have exhibited alterations and appear to be subject to regulation by miRNAs in the context of CRC [29] (summarized in Table 1 and Figure 3).

### 5.1. Wnt/β-catenin Pathway

The Wnt/β-catenin signaling pathway is a central driver of colorectal carcinogenesis, primarily activated through mutations in the APC gene. These mutations lead to the nuclear localization of β-catenin, resulting in the upregulation of Wnt target genes, which promote tumor progression [30]. Emerging research implicates specific miRNAs, particularly the miR-17-92 cluster, in modulating the Wnt/β-catenin pathway and influencing CRC progression.

The miR-17-92 cluster is significantly influenced by the APC/β-catenin pathway in CRC. It is downregulated in response to APC mutations and upregulated when β-catenin expression is enforced. Additionally, activated β-catenin due to APC mutations directly activates the miR-17-92 promoter. Enforcing miR-19a expression can override APC’s tumor-suppressive effects, while knocking down miR-19a in cancer cells with compromised APC function reduces their aggressiveness in vitro. High miR-19a expression correlates with elevated β-catenin levels and advanced tumor stages in CRC specimens, suggesting that targeting the miR-17-92 cluster may have therapeutic potential in colon cancers with abnormal APC/β-catenin signaling. This mechanism elucidates how APC mutations impact both the Wnt/β-catenin pathway and miR-17-92 cluster expression, contributing to CRC progression [31].

Furthermore, the role of the miR-17-92 cluster in CRC is multifaceted, with its members exerting different effects depending on their expression levels. Colon cancer progression is influenced by miR-17-92 cluster expression levels. Moderate miR-19a levels within the cluster induce tumor metastasis via a Wnt/β-catenin-mediated EMT by targeting the tumor suppressor PTEN. In contrast, higher miR-17-92 levels shift their targeting from PTEN to oncogenes, including Ctnnb1 (β-catenin) through miR-18a, inhibiting tumor growth and metastasis. Elevating Ctnnb1 in high-miR-17-92 cells does not raise β-catenin protein levels, suggesting that other factors regulated by miR-17-92 contribute to tumor inhibition, potentially negatively affecting β-catenin production. Thus, elevated miR-17-92 levels critically suppress the Wnt/β-catenin pathway, holding potential therapeutic implications [32].

Another pivotal player in CRC progression is MIR17HG, an RNA gene encoding the miR-17-92 cluster. Elevated MIR17HG expression in colon cancer significantly correlates with lymph node metastasis and TNM stage, and serves as an independent prognostic indicator for overall and disease-free survival. The suppression of MIR17HG hampers colon cancer cell viability, invasion, and the EMT process, impairing in vivo tumor formation. MIR17HG knockdown leads to the downregulation of miR-17 and miR-18a. Additionally, the oncogenic role of MIR17HG in colon cancer relies on activating the Wnt/β-catenin signaling pathway through upregulating miR-17 and miR-18a, further promoting disease progression [33].

Furthermore, as mentioned earlier, miR-92a upregulation has been observed in chemoresistant CRC cells and tissues, where it confers resistance to chemotherapy-induced apoptosis. miR-92a’s oncogenic activity is mediated through the direct targeting of KLF4, GSK3β, and DKK3, which are negative regulators of the Wnt/β-catenin pathway. Additionally, the IL-6/STAT3 pathway has been shown to increase miR-92a expression by directly targeting its promoter. This activation of the Wnt/β-catenin pathway by miR-92a promotes stem-like phenotypes in CRC cells, underscoring its significance in CRC progression [28].

Another study has identified miR-17-5p as an oncogenic miRNA that coordinates tumorigenesis and progression by targeting the P130-encoding gene, subsequently activating the Wnt/β-catenin pathway. Elevated miR-17-5p expression in colorectal cancer patient cohorts correlates with shorter overall survival. Paradoxically, it also correlates with a more favorable response to adjuvant chemotherapy compared to patients with low miRNA expression. A robust inverse correlation exists between miR-17-5p levels and P130 expression. These findings underscore the pivotal role of miR-17-5p as a determinant in colorectal cancer progression [34].

Moreover, miR-19a-3p and its interaction with FOXF2 have been implicated in CRC. High serum levels of miR-19a-3p and low levels of FOXF2 correlate with various clinical parameters in CRC patients. Silencing miR-19a-3p and overexpressing FOXF2 suppress the EMT, invasion, migration, and proliferation of CRC cells. Mechanistically, miR-19a-3p directly targets FOXF2, and its inhibition leads to upregulation of the FOXF2-mediated Wnt/β-catenin signaling pathway, affecting various cellular processes [35].

Metabolic CRC presents a unique challenge, and a study has identified miR-20a as a key player in this context. miR-20a is highly expressed in metabolic CRC but minimally in normal colorectal tissues. This miRNA has been associated with dysregulated events, including the Wnt signaling pathway and enzymes involved in fatty acid metabolism. miR-20a’s upregulation promotes CRC cell proliferation and migration by upregulating fatty acid synthesis enzymes through the Wnt/β-catenin signaling pathway. Targeting miR-20a could offer a potential strategy for preventing tumor metastasis in metabolic CRC [36].

Additionally, radio-resistance is a significant challenge in CRC treatment. Exosomal miR-19b has been implicated in CRC radio-resistance. The overexpression of miR-19b in CRC tissues is associated with a poor prognosis. CRC-derived exosomes (EXOs) enhance radio-resistance and stemness properties in CRC cells by delivering miR-19b. Furthermore, miR-19b targets FBXW7, and the reintroduction of FBXW7 reverses the effects of miR-19b on radio-resistance and stemness. The Wnt/β-catenin pathway has been implicated in this process, further highlighting the role of Wnt signaling in CRC progression and therapy [37].

In conclusion, the intricate interplay between the miR-17-92 cluster and the Wnt/β-catenin signaling pathway plays a central role in colorectal cancer progression. Understanding these regulatory networks provides insights into potential therapeutic targets and strategies for effectively managing this deadly disease. Future research in this field may lead to innovative approaches for CRC treatment and improved patient outcomes.

### 5.2. Epidermal Growth Factor Receptor Signaling Pathway

The epidermal growth factor receptor (EGFR), a transmembrane glycoprotein belonging to the human EGFR family, plays a pivotal role in cellular physiology [38]. Upon activation, it initiates intracellular signaling by engaging two major subnetworks: the KRAS/RAF/MEK and PI3K/AKT pathways [38]. This well-characterized pathway critically modulates various aspects of human cell biology, including survival, proliferation, migration, angiogenesis, and apoptosis, and is implicated in several epithelial cancers, including CRC [39]. Research efforts in this domain have illuminated the extensive involvement of miRNAs in the regulation of EGFR signaling, thereby enhancing our comprehensive understanding of intestinal carcinogenesis [40].

The AKT–PI3K–mTOR/PTEN axis represents the secondary signaling hub within the EGFR pathway, exhibiting amplification in nearly 20% of CRC cases [41]. In both physiological contexts and malignancy, multiple oncomirs and tumor-suppressor miRNAs intricately interact and modulate the functionality of this cascade. For instance, the miR-17-92 cluster directly regulates the 3′UTR region of the PTEN gene, resulting in aberrant cell proliferation and atypical angiogenesis [42]. Furthermore, miR-21, miR-19, and miR-96, prominent oncogenes, have also been implicated in CRC carcinogenesis through their role in stimulating the AKT/PI3K pathway [43].

### 5.3. Extracellular Matrix Breakdown and Epithelial–Mesenchymal Transition

Transglutaminase-2 (TG2) plays a crucial role in extracellular matrix (ECM) cross-linking within the tumor microenvironment (TME). While its association with colorectal cancer is established, its functional role appears to be context-dependent. miR-19 was identified as a regulator of TG2 through in silico analysis and confirmed experimentally. TG2 expression inversely correlated with invasion; knockdown in SW480 cells increased invasion, while overexpression in SW620 cells had the opposite effect. TG2 expression was present in primary colorectal tumors but absent in liver metastases. Additionally, miR-19 overexpression and consequent TG2 reduction were linked to chromosome-13 amplification events, altering invasive behavior in colorectal cancer cells [44].

### 5.4. TGF-β Signaling Pathway

The TGF-β/Smad molecular pathway plays a crucial role in cell proliferation and is closely linked to the invasiveness and metastatic potential of cancer. In the context of CRC, this pathway exhibits a dual role, acting as a tumor suppressor in the early stages of the disease while simultaneously functioning as a potent cancer promoter in advanced neoplasms [45].

Numerous microRNAs have been identified as regulators of TGFβ receptor 2 (TGFBR2), a key component of the signaling process [46]. In addition to TGFBR2, Smad-4 is a vital mediator in the TGF-β signaling cascade, and its disruption results in distal metastases and generally a poor prognosis. miRNA-20-5p has been found to significantly silence the Smad gene, leading to increased proliferation and cancer invasiveness [47].

## 6. The Role of miR-17-92 in Colorectal Tumorigenesis

### 6.1. Tumor Initiation and Growth

The elevated expression of the miR-17-92 cluster has emerged as a pivotal contributor to the promotion of cell proliferation and the inhibition of apoptosis, substantiating its significant role in the initiation and progression of CRC tumors (summarized in Table 1) [7]. An investigation has elucidated the mechanistic underpinning of this phenomenon, highlighting the direct targeting of the anti-apoptotic molecule BCL-2-interacting mediator of cell death (BIM) by miR-92a within colon cancer tissues. Notably, the introduction of an anti-miR-92a antagomir yielded the induction of apoptosis in colon-cancer-derived cell lines, reinforcing the critical involvement of miR-92a in the pathogenesis of colorectal carcinoma [7]. Another study underscores the substantial role of miR-17-5p, classified as an oncofetal miRNA, as a key determinant in the regulation of colorectal cancer progression. miR-17-5p, identified as an oncogenic miRNA, exerts its regulatory influence on tumorigenesis and the progression of CRC through the precise targeting of the gene encoding P130, consequently activating the Wnt/β-catenin signaling pathway. Extensive clinical analyses, encompassing two expansive cohorts of colorectal cancer patients, have revealed intriguing observations. Patients characterized by heightened expression levels of miR-17-5p within their tumor specimens exhibited notably shortened overall survival rates. Paradoxically, these individuals displayed a more favorable response to adjuvant chemotherapy when compared to their counterparts harboring tumors with diminished miRNA expression. Furthermore, a robust inverse correlation has been discerned between miR-17-5p and P130 expression levels. Collectively, these contemporary findings underscore the critical and multifaceted role of miR-17-5p as a determinative factor in the progression of colorectal cancer, holding implications for prognostic evaluation and therapeutic strategies [34].

There has been shown a significant increase in the proliferation and migration capacity of both SW620 and LoVo cells following miR-92a mimic transfection, whereas miR-92a inhibition reduced these effects. Furthermore, bioinformatics and luciferase reporter analysis revealed KLF4 as a direct target of miR-92a in CRC cells. Overexpressing KLF4 mitigated miR-92a’s impact on CRC cell motility. Subsequent investigations revealed aberrant downregulation of the cell cycle inhibitor p21 in CRC cells, a condition reversed by miR-92a inhibition. This suggests that miR-92a may promote colorectal carcinogenesis by enhancing CRC cell proliferation and migration via KLF4 and downstream p21 regulation, offering a potential therapeutic target for CRC [48].

### 6.2. Stimulation of Angiogenesis

Angiogenesis, the process of forming new blood vessels, is critical for tumor growth and survival by providing nutrients and oxygen to cancer cells [49]. The miR-17-92 cluster can indirectly promote angiogenesis by targeting and suppressing the expression of anti-angiogenic factors like thrombospondin-1 (TSP-1) [5]. The miR-17-92 cluster has demonstrated its ability to suppress tumors by effectively inhibiting tumor angiogenesis in a mouse model that was genetically engineered. miR-17-92 exerts its inhibitory effect on the progression of CRC by reducing tumor angiogenesis which is achieved by targeting several tumor angiogenesis-inducing genes, including TGFBR2, HIF1α, and VEGFA, both in vivo and in vitro [50].

Mutations in the KRAS and Myc proto-oncogenes, along with alterations in the TP53 tumor suppressor gene, are common in human adenocarcinomas. These genetic anomalies often promote angiogenesis by maintaining vascular endothelial growth factor (VEGF) production. However, when mouse colon cells altered with KRAS and lacking p53 were observed, they developed slow-growing tumors with limited vascularization. This contrasted with cells where Myc was introduced via a retrovirus, resulting in significantly increased tumor vascularization and growth. Interestingly, Myc’s presence did not affect VEGF levels directly. Instead, it was associated with the reduced expression of anti-angiogenic factors like thrombospondin-1 (Tsp1) and connective tissue growth factor (CTGF). The miR-17-92 cluster, known to suppress Tsp1 and CTGF and found to be elevated in colon cells expressing both KRAS and c-Myc, seems to play a role here. When miR-17-92 was inhibited using antisense 2′-O-methyl oligoribonucleotides, there was a partial reversion in the expression of Tsp1 and CTGF. Additionally, introducing an miR-17-92-bearing retrovirus into cells only expressing Ras led to decreased levels of Tsp1 and CTGF. Cells treated in this manner formed tumors that were larger and had a better blood supply. These results highlight the significant influence of microRNAs in shaping Myc-induced tumor characteristics beyond the cellular level [5].

Extracellular vesicles (EVs) are nanometer-sized membranous vesicles that facilitate basic intercellular communication. An earlier study showed that EVs originating from colon cancer cells are rich in miR-92a-3p and promote angiogenesis. Although the identified target is Dickkopf-3 (DKK3), its pro-angiogenic effects are not solely due to the suppression of DKK3. The study reveals that introducing miR-92a-3p into endothelial cells leads to the increased expression of genes related to cell cycle and mitosis, and the decreased expression of genes associated with cell adhesion. Additionally, the study discovered a new target of miR-92a-3p, claudin-11, a member of the claudin family critical for forming tight junctions (TJs) in cells. The disruption of TJs and the resultant loss of claudin expression are crucial steps in the epithelial-to-mesenchymal transition. This study uncovers a novel mechanism by which EVs from tumors can induce a partial endothelial-to-mesenchymal transition in endothelial cells, contributing to tumor angiogenesis [51].

Numerous studies have highlighted the varied roles of DKK3 in tumor angiogenesis and oncogenesis [52,53,54,55]. DKK3 promotes angiogenesis by upregulating VEGF [56]. Increased DKK3 expression in CRC tissue compared to adjacent normal tissue is associated with elevated microvessel formation [57]. Moreover, DKK3 modulates the Wnt/β-catenin signaling pathway and holds promise as a diagnostic and prognostic biomarker in the serum of CRC patients [58].

### 6.3. Metastasis

Dysregulation of miR-17-92 has been associated with an increased likelihood of metastasis in CRC. It can enhance the invasive and migratory abilities of cancer cells, facilitating their spread to distant organs. Numerous studies have substantiated the significant role of miR-92a in facilitating the invasion and migration of CRC via the RECK-MMP signaling pathway, with a concomitant increase in miR-92a levels being closely correlated with an unfavorable long-term prognosis in CRC [59]. Recent findings from a meta-analysis have underscored the pronounced associations between several miRNA clusters, including miR-17/92a-1, miR-106a/363, miR-106b/93/25, and miR-183/96/182, and the incidence of metastasis as well as the diminished survival rates of patients, positioning them as particularly promising targets for translational research [60]. Moreover, a subsequent investigation has unveiled compelling evidence linking heightened expression levels of miR-17-3p and miR-92a in the invasive front of CRC to early regional lymph node metastasis (LNM) [61]. These findings collectively corroborate the pivotal role of the miR-17/92 cluster in the initial stages of CRC metastatic progression, prompting a call for further exploration in this realm.

Furthermore, it has been discerned that elevated expression levels of circulating exosomal miR-17-5p and miR-92a-3p exhibit significant associations with the pathological stages and grades of CRC patients [62]. These circulating exosomal miRNAs hold promise as non-invasive prognostic biomarkers for both primary and metastatic CRC. Additionally, an investigation has established a correlation between miR-92a overexpression and specific bio-pathological features of colorectal cancer, encompassing TNM stage, lymph node and distant metastases, as well as overall patient survival [63]. Consequently, miR-92a emerges as a potential molecular prognostic marker for CRC and its disease progression.

Furthermore, novel insights into the role of miR-20a in CRC have surfaced, elucidating its status as an independent prognostic factor for CRC patients [64]. This study has provided a mechanistic basis for the dysregulation of Smad-4 and its contributions to CRC cell migration, invasion, and the EMT, thereby establishing miR-20a as a plausible oncogenic factor in CRC with potential therapeutic implications [64].

Similarly, the elevated expression of miR-17 has been implicated in liver metastasis in CRC, prompting a need for an in-depth exploration of its downstream pathways to unravel the mechanisms underlying this phenomenon [65]. Notably, a strong positive correlation between miR-92a expression in tumor tissues and lymph node metastasis in CRC patients has been established, and this correlation remained significant after adjustments for age, sex, and disease differentiation. Conversely, a negative correlation between miR-92a levels and the PTEN gene was identified, with no significant association detected between miR-92a and E-cadherin. Patients exhibiting high miR-92a/low PTEN levels exhibited notably poorer overall survival and disease-free survival rates than those with high miR-92a/high PTEN, low miR-92a/high PTEN, or low miR-92a/low PTEN. Additionally, the influence of miR-92a and PTEN levels on tumor cell migration in CRC was corroborated through experimentation with CRC cell models, thereby implicating miR-92a in the lymph node metastasis of CRC patients via the PTEN-regulated PI3K/AKT signaling pathway [66]. These studies collectively contribute to a comprehensive understanding of miR-17-92 cluster involvement in CRC progression and metastasis, offering potential avenues for further research and the development of novel diagnostic and therapeutic strategies for CRC patients.

### 6.4. Involvement in Epithelial–Mesenchymal Transition (EMT)

The miR-17-92 cluster has been implicated in the regulation of the EMT in CRC and other types of cancer. The EMT refers to a phenomenon where epithelial cells undergo a change in characteristics, acquiring mesenchymal traits. This change leads to increased cell movement, invasiveness, and resistance to apoptosis. Notably, the EMT plays a critical role in cancer metastasis.

It has been shown that the levels of miRNA-17-5p expression were significantly lower in primary CRC tissues with signs of metastasis compared to those without metastatic features. Subsequently, there was an inverse relationship between miRNA-17-5p expression and vimentin across five different CRC cell lines. Furthermore, the overexpression of miRNA-17-5p led to a decrease in vimentin expression along with reduced cell migration and invasion abilities, in both LoVo and HT29 cell lines [67]. On the other hand, inhibiting miRNA-17-5p showed the opposite effect. Through experiments involving Ago2 immunoprecipitation and luciferase assays, this study demonstrated a binding interaction between miRNA-17-5p and the 3′ untranslated region (3′UTR) of VIM mRNA. Additionally, other studies have revealed that miRNA-17-5p can inhibit the spread of CRC to the liver [67].

Similarly, cancer-associated fibroblasts (CAFs) play a role in promoting the EMT, metastasis, and resistance to chemotherapy in CRC cells. Notably, CAFs achieve these roles by transferring exosomes to CRC cells, which leads to a significant increase in levels of miR-92a-3p within those cells. Mechanistically, the higher expression of miR-92a-3p activates the Wnt/β-catenin pathway while simultaneously suppressing apoptosis by directly inhibiting FBXW7 and MOAP1. These molecular changes collectively contribute to enhanced cell stemness, the EMT, metastasis, and resistance against 5 FU/L-OHP treatment in CRC cases. Importantly, clinical analyses have supported these findings by demonstrating increased expression of miR-92a-3p in CRC tissues. Furthermore, this elevated miR-92a-3p expression is inversely correlated with levels of FBXW7 and MOAP1 in CRC samples, highlighting the significance of these interactions [68]. Overall, miR-17-92 is a complex regulator of the EMT in colorectal cancer, and its dysregulation can impact the balance between epithelial and mesenchymal characteristics in CRC cells. Further research is needed to fully elucidate the mechanisms and clinical implications of miR-17-92-mediated EMT regulation in CRC.

### 6.5. Role of miR-17-92 in Responsiveness and Resistance to Therapy

The dysregulation of miR-17-92 in CRC has been implicated in conferring resistance to chemotherapy and targeted therapies, potentially through its regulatory influence on genes associated with drug response and apoptosis. Notably, an elevation in the expression level of miRNA-17-5p has been observed in chemoresistant CRC patients. Moreover, CRC patients presenting with distant metastases and advanced clinical stages exhibit significantly higher levels of miR-17-5p. A Kaplan–Meier survival analysis reveals that patients with elevated miR-17-5p levels experience diminished survival rates, particularly among those who have previously undergone chemotherapy. In vitro investigations have demonstrated that the overexpression of miR-17-5p promotes invasiveness in COLO205 cells. Furthermore, the study established PTEN as a direct target of miR-17-5p in colon cancer cells, with their context-specific interactions being implicated in the development of multidrug resistance. It is noteworthy that chemotherapy interventions have been shown to elevate miR-17-5p expression levels, thereby further repressing PTEN and contributing to the onset of chemoresistance [42].

Furthermore, an in-depth exploration into the mechanisms underlying miR-17-5p-mediated chemotherapy resistance reveals that its overexpression leads to reduced apoptosis and decreased drug sensitivity both in vitro and in vivo, particularly under 5-fluorouracil (5-FU) treatment conditions. This phenomenon strongly suggests that miR-17-5p plays a pivotal role in mediating resistance to 5-FU chemotherapy. Bioinformatic analyses have uncovered an association between miR-17-5p-mediated chemoresistance and mitochondrial homeostasis. Specifically, miR-17-5p directly targets the 3′ untranslated region of mitofusin 2 (MFN2), resulting in reduced mitochondrial fusion and heightened mitochondrial fission and mitophagy. Notably, the downregulation of methyltransferase-like protein 14 (METTL14) in CRC has been observed, leading to decreased m6A levels. This reduction in METTL14 levels subsequently enhances the expression of pri-miR-17 and miR-17-5p, adding another layer to the intricate regulatory network governing chemoresistance in CRC [69]. Further study showed that 5-FU diminishes c-Myc expression, subsequently leading to a decrease in the miR-17-92 cluster and an increase in TSP-1 mRNA expression [70].

The expression levels of five miRNAs, specifically miRNA223-3p, miRNA20a-5p, miRNA17-5p, miRNA19a-3p, and miRNA7-5p, were quantified in the peripheral blood of 77 CRC patients, along with the expression of three proteins, PTEN, ERK, and EGFR. Higher levels of circulating miRNAs had been observed in CRC patients compared to healthy controls at baseline. These levels had been reduced after 3 months of 5-FU-based therapy and then significantly increased after 6 months in responder patients compared to non-responders. miRNA19a-3p exhibited a noteworthy pattern of change in subgroups of patients with high ERK, EGFR, and PTEN protein levels, and its level after 6 months of 5-FU-based therapy had significant implications for the hazard of increased risk of disease recurrence and progression [71].

**Table 1:** Summary of miR-17-92 cluster main targets and the consequent oncosuppressive effect in CRC.

miRNA	Dysregulation	Targets	Pathway/Effects	Experimental Validation	References
miR-19a	Upregulation	β-catenin/APC mutation	Wnt/β-catenin pathway	Luciferase reporter vectors and protein expression changes	[31]
miR-19a	Moderate	PTEN		Luciferase assays	[32]
miR-92a	Upregulation	KLF4, GSK3β, and DKK3	Negative regulators of the Wnt/β-catenin pathway	Luciferase assays	[28]
miR-17-5p		P130	Activating the Wnt/β-catenin pathway	Luciferase assays	[34]
miR-19a-3p	Upregulation	FOXF2	Increase EMT, invasion, migration, and proliferation of CRC cells Activating the Wnt/β-catenin pathway	Dual luciferase reporter assay	[35]
miR-20a	Upregulation	-	metabolic CRC Activating the Wnt/β-catenin pathway	-	[36]
miR-19b	Upregulation	FBXW7	Radio-resistance and stemness properties	Dual luciferase reporter assay	[37]
miR-21, miR-19, and miR-96	Upregulation	-	AKT/PIK3 pathway	-	[43]
microRNA-20–5p	Upregulation	Smad-4	TGF-b signaling pathway	Luciferase reporter assay	[47]
miR-92a	Upregulation	BCL-2-interacting mediator of cell death (BIM)	Promotion of cell proliferation and the inhibition of apoptosis	Protein expression changes	[7]
miR-92a	Upregulation	KLF4, p21,	Increase in the proliferation and migration capacity	Bioinformatics and luciferase reporter analysis and protein expression changes	[48]
miR-17-92	Upregulation	TGFBR2, HIF1α, and VEGFA	Angiogenesis	-	[50]
miR-17-92	Upregulation	suppress Tsp1 and CTGF	Angiogenesis	Protein expression changes	[5]
miR-92a-3p	Upregulation	DKK3 and claudin-11	Promote angiogenesis	Luciferase reporter assay	[51]
miR-92a-3p	Upregulation	-	RECK-MMP signaling pathway increase invasion and migration	-	[59]
miR-17-3p and miR-92a	Upregulation	-	Metastasis	-	[61,62]
miRNA-17 -5p	Down-regulation	vimentin	EMT	Luciferase reporter vectors and protein expression changes	[67]
miR-92a-3p	Upregulation	FBXW7 and MOAP1	Enhanced cell stemness, EMT, metastasis and resistance against 5 FU/L-OHP treatment	Luciferase report assay, real-time qPCR, Western blot	[68]
miR-17-5p	Upregulation	MFN2 METTL14 l	5-FU chemotherapy resistance reduced apoptosis and decreased drug sensitivity	Luciferase reporter as-say	[69]
miRNA20a-5p, miRNA17-5p, miRNA19a-3p	Upregulation	PTEN, ERK, and EGFR	5-FU chemotherapy responsive	Protein expression level changes	[71]

## 7. miR-17-92 as a Diagnostic and Prognostic Biomarker

### 7.1. Utility of miR-17-92 as an Early Diagnostic Tool for CRC

The potential of circulating miRNAs as diagnostic biomarkers is highly promising. Studies consistently demonstrate that miRNAs exhibit distinct signatures specific to various cancer subtypes, including breast, prostate, lung, and colorectal cancers. Notably, these miRNAs remain stable within the body and have been detected in various bodily fluids such as saliva, urine, plasma, serum, tears, and breast milk [72]. Their stability is attributed to their encapsulation within microvesicles and exosomes, as well as their association with argonaute proteins, which shield them from degradation [73]. A comprehensive meta-analysis of 34 scientific studies revealed that assays conducted on serum or plasma consistently produced reliable outcomes, with a 76% rate of sensitivity and specificity for a single altered miRNA signature. These studies pinpointed 28 miRNAs as potential biomarkers, highlighting the potential of miRNAs as diagnostic and prognostic tools for colorectal cancer detection [74]. Circulating miRNAs not only serve as highly effective biomarkers for identifying various cancer subtypes but are also valuable for monitoring tumor metastasis and progression, indicating responsiveness to clinical treatments, offering substantial prognostic value in the disease, and demonstrating a high level of sensitivity [75].

### 7.2. Predictive Value of miR-17-92 Expression in CRC Prognosis

It has been observed that initial levels of miRNA-92a exhibited greater efficacy than carcinoembryonic antigen levels in diagnosing 44 patients with metastatic colorectal cancer (mCRC) when compared to 17 healthy individuals. Additionally, patients who eventually succumbed to the disease displayed elevated levels of miRNA-92a and miRNA-222 at the 24-week mark. However, a multivariate Cox analysis revealed that higher miRNA-222 levels at 24 weeks were associated with reduced overall survival. These findings collectively suggest that extracellular vesicle (EV)-miRNAs hold significant promise as liquid biopsy markers for detecting and predicting the progression of mCRC [76]. Another study conducted an miRNA microarray analysis that identified significant differences in miRNA expressions between chemoresistant and chemosensitive patients with CRC. Among these miRNAs, six (miR-100, miR-92a, miR-16, miR-30e, miR-144-5p, and let-7i) were significantly dysregulated and showed potential as biomarkers for distinguishing between chemoresistant and chemosensitive CRC patients. These miRNAs demonstrated no correlation with tumor location, stage, or chemotherapy regimen, except for miR-100, which was upregulated in lower histological grades. Gene ontology and KEGG pathway analyses linked these miRNAs to key signaling pathways, further emphasizing their potential as biomarkers for monitoring chemotherapy responses and as targets for CRC treatment [77].

## 8. The Role of Circulating the miR-17-92 Cluster in Plasma and Serum for CRC Diagnosis

An extensive study explores the potential of miRNAs as a molecular diagnostic tool for cancer, with a specific focus on CRC. The investigation delves into the role of circulating miRNAs in plasma and serum for cancer diagnosis, with a particular emphasis on their capacity to serve as biomarkers for early CRC detection. The study examined twelve miRNAs in plasma samples from patients with advanced colorectal neoplasia, encompassing carcinomas and advanced adenomas, as well as healthy controls, utilizing real-time RT-PCR. Notably, miR-29a and miR-92a emerged as particularly significant in diagnosing advanced neoplasia. Specifically, miR-29a and miR-92a exhibited areas under the ROC curve (AUC) of 0.844 and 0.838, respectively, in distinguishing CRC from control subjects. These miRNAs also proved effective in discerning advanced adenomas from controls, with miR-29a displaying an AUC of 0.769 and miR-92a an AUC of 0.749. When combined in ROC analyses, these two miRNAs achieved an AUC of 0.883 with 83.0% sensitivity and 84.7% specificity for identifying CRC and an AUC of 0.773 with 73.0% sensitivity and 79.7% specificity for advanced adenomas. These findings underscore the potential of miR-29a and miR-92a as non-invasive biomarkers for the early detection of CRC [78]. Another study analyzed serum samples from 200 CRC patients, 50 advanced adenoma patients, and 80 healthy controls, measuring the levels of five miRNAs (miR-21, miR-31, miR-92a, miR-18a, and miR-106a) using real-time quantitative polymerase chain reaction (RT-PCR). The study revealed significant elevations in miR-21 and miR-92a levels in both CRC and advanced adenoma patients compared to healthy controls. Specifically, miR-21 and miR-92a displayed areas under the receiver operating characteristics (ROC) curve of 0.802 and 0.786, respectively, for discriminating CRC from controls and 0.709 and 0.701 for advanced adenomas. A combined ROC analysis of these two miRNAs improved the AUC to 0.847 for CRC and 0.722 for advanced adenomas. Notably, high miR-92a expression in CRC was associated with poorer survival outcomes in a multivariate Cox proportional hazards analysis. However, no significant differences were observed in the levels of miR-18a, miR-31, and miR-106a among the groups. This study suggests that serum levels of miR-21 and miR-92a could serve as valuable early detection markers for CRC, with miR-92a also providing prognostic insights into CRC patient outcomes [79].

## 9. Fecal miR-17-92 Cluster for CRC Diagnosis

Several studies have explored the potential of miRNAs as diagnostic biomarkers for CRC. In one study, 11 fecal miRNAs were analyzed in CRC tissue and normal mucosa. All miRNAs exhibited significantly higher expression in cancer tissue compared to normal mucosa. Additionally, five of these miRNAs (miR-19-b-3p, miR-20a-5p, miR-21-3p, miR92a-3p, and miR141) displayed significantly elevated levels in CRC patients compared to controls before surgery, but their expression decreased significantly after curative surgery. This suggests that these three specific fecal miRNAs could serve as promising markers for CRC secondary prevention. Nevertheless, further investigation in large prospective trials is necessary to confirm their utility and compare them with existing screening tools [80]. Another study observed that miR-221 and miR-18a were upregulated in CRC and exhibited significantly higher levels in CRC tumors compared to adjacent normal tissues. Stool samples showed a significant increase in miR-221 and miR-18a levels from normal controls to late-stage CRC. Both miRNAs were significantly elevated in early (stages I + II) and advanced (stages III + IV) CRC, with AUC values of 0.73 and 0.67 for CRC detection. No differences were observed between proximal and distal CRC, and antibiotic use did not affect miRNA levels [81]. These findings suggest that miR-221 and miR-18a may serve as potential biomarkers for the early detection of CRC. Additionally, miR-20a was significantly upregulated in CRC tumors and in fecal samples from CRC patients. Its diagnostic potential was highlighted with an AUROC of 0.73, sensitivity of 55%, and specificity of 82% for CRC detection. No variation was noted between cancer locations, and antibiotic use did not affect fecal miR-20a levels, suggesting it as a promising non-invasive biomarker for CRC screening [82]. Furthermore, the combination of miR-223 and miR-92a in fecal and plasma samples demonstrated a high sensitivity of 96.8% and specificity of 75% for CRC detection. This two-miRNA biosignature in CRC clinical specimens offers a promising method for sensitive CRC detection [83]. Lastly, the miR-17-92 cluster and miR-135 exhibited significantly higher expression in CRC tissues compared to normal tissues, while miR-21 levels remained relatively consistent. Analyzing exfoliated colonocytes from 197 CRC patients and 119 healthy individuals revealed a sensitivity of 74.1% and specificity of 79.0% for CRC detection. Sensitivity was influenced by tumor location. This suggests that miRNA analysis of isolated colonocytes from feces could be a valuable CRC screening method, warranting further exploration of oncogenic miRNA as potential screening markers [84]. Among eight tested miRNAs, miR-21, miR-92a, miR-144*, and miR-17-3p had significantly different stool levels between CRC patients and controls. Sensitivities and specificities for these miRNAs were 79.3%/48.3%, 89.7%/51.7%, 78.6%/66.7%, and 67.9%/70.8%, respectively. In a multivariate analysis, miR-92a and miR-144* were significantly associated with CRC presence. These miRNAs show promise as non-invasive biomarkers for CRC detection [85]. In this study, 11 miRNAs were analyzed in CRC tissue and normal mucosa. All miRNAs were significantly upregulated in cancer tissue. Among the five fecal miRNAs studied (miR-19-b-3p, miR-20a-5p, miR-21-3p, miR92a-3p, and miR141), CRC patients showed higher preoperative levels, which decreased after surgery. Three of these miRNAs (miR20a-5p, miR21-3p, and miR141) returned to normal levels post-surgery [86]. Collectively, these findings highlight the potential of miRNAs, both individually and in combination, as non-invasive biomarkers for CRC screening and early detection. Further research and larger-scale prospective trials are essential to confirm their clinical utility and compare them with existing screening tools.

## 10. Conclusions

In the realm of colorectal cancer research, the role of the miR-17-92 cluster has emerged as a multifaceted and pivotal contributor to various aspects of tumorigenesis and progression. This miR-17-92 cluster, comprised of several individual miRNAs, exerts its influence on tumor initiation, angiogenesis, metastasis, and the EMT. The diverse functions of miR-17-92 in colorectal cancer underscore its significance as a potential diagnostic, prognostic, and therapeutic target [87].

Firstly, in tumor initiation and growth, miR-17-92 has been shown to promote cell proliferation and inhibit apoptosis, contributing to the initiation and progression of colorectal cancer tumors. Specific miRNAs within the cluster, such as miR-92a and miR-17-5p, target critical genes involved in colorectal carcinogenesis, highlighting their potential as therapeutic targets and prognostic markers.

Secondly, the cluster plays a substantial role in stimulating angiogenesis, a critical process for tumor growth and survival. By targeting anti-angiogenic factors like TSP-1 and regulating key angiogenic genes, miR-17-92 indirectly promotes the formation of new blood vessels in the tumor microenvironment.

Moreover, miR-17-92′s involvement in metastasis is notable. The dysregulation of miR-92a and other cluster members enhances the invasive and migratory abilities of CRC cells, leading to an increased likelihood of metastasis. The cluster’s influence on the tumor microenvironment and its correlation with clinical outcomes make it a promising avenue for further research.

Lastly, the regulation of the EMT by miR-17-92 adds another layer to its complexity in CRC. This microRNA cluster can impact the balance between epithelial and mesenchymal characteristics in CRC cells, affecting their mobility and invasiveness, which are key factors in metastasis.

The dysregulation of miR-17-92 in CRC presents a complex and multifaceted landscape that has significant implications for the diagnosis, prognosis, and treatment of the disease. This microRNA cluster plays a crucial role in determining responsiveness and resistance to therapy in CRC patients. By influencing genes associated with drug response and apoptosis, miR-17-92 can confer resistance to chemotherapy and targeted therapies. The elevated expression of miR-17-5p has been observed in chemoresistant CRC patients, leading to decreased survival rates, particularly in those who have undergone chemotherapy. The cluster’s role in mediating resistance to chemotherapy, particularly 5-FU, has been linked to mitochondrial homeostasis, further adding complexity to the chemoresistance network in CRC.

Moreover, miR-17-92 shows promise as both a diagnostic and prognostic biomarker for CRC. Studies have explored the potential of circulating miRNAs in plasma/serum and fecal samples as non-invasive tools for the early detection and monitoring of CRC. miR-17-92 cluster members, such as miR-92a and miR-20a, have demonstrated significant diagnostic potential for distinguishing between CRC patients and healthy controls. Additionally, these miRNAs offer insights into predicting patient responses to treatment and the risk of disease recurrence and progression.

## 11. Future Perspectives

The study of miR-17-92 in colorectal cancer is a dynamic field with several avenues for future research and potential clinical applications; miR-17-92 and its specific miRNAs, such as miR-92a, offer potential therapeutic targets for the development of novel CRC treatments. Further research into targeted therapies, including anti-miRNA strategies, may yield promising results. The expression levels of miR-17-92 cluster members could serve as diagnostic and prognostic biomarkers for colorectal cancer. Continued investigation into their clinical significance and validation in large patient cohorts is essential. Understanding the mechanisms by which miR-17-92 promotes metastasis and the EMT in CRC can lead to the development of therapies aimed at inhibiting these processes, ultimately improving patient outcomes. Exploring the potential synergy between miR-17-92-targeted therapies and existing CRC treatments, such as chemotherapy or immunotherapy, could enhance the effectiveness of current treatment modalities. Tailoring treatment strategies based on the specific miRNA expression profiles of CRC patients could lead to more personalized and effective therapeutic approaches.

The role of miR-17-92 in CRC therapy responsiveness and resistance opens up several avenues for future research and clinical applications; further investigation into miR-17-92 and its impact on drug response could lead to the development of personalized treatment strategies. Tailoring therapies based on the miRNA profile of CRC patients may enhance treatment effectiveness. Developing therapies aimed at modulating miR-17-92 expression or activity could help overcome chemoresistance and improve treatment outcomes for CRC patients. Large-scale prospective trials are needed to validate the diagnostic and prognostic potential of miR-17-92 and other miRNAs in CRC. Comparing their performance with existing screening tools will be crucial for their clinical utility. Investigating the utility of combining multiple miRNAs or miRNA panels with other established CRC biomarkers may enhance the accuracy of early detection and prediction of treatment response. Further research into the underlying mechanisms through which miR-17-92 contributes to chemoresistance and its impact on mitochondrial homeostasis may unveil novel therapeutic targets. Combining miRNA analysis with advanced imaging techniques may provide a comprehensive approach to CRC diagnosis and treatment monitoring.

## Data Availability

Not applicable.

## References

[1] Bray F., Ferlay J., Soerjomataram I., Siegel R.L., Torre L.A., Jemal A. (2018). Global cancer statistics 2018: GLOBOCAN estimates of incidence and mortality worldwide for 36 cancers in 185 countries. CA Cancer J. Clin..

[2] Rattray N.J.W., Charkoftaki G., Rattray Z., Hansen J.E., Vasiliou V., Johnson C.H. (2017). Environmental influences in the etiology of colorectal cancer: The premise of metabolomics. Curr. Pharmacol. Rep..

[3] He L., Thomson J.M., Hemann M.T., Hernando-Monge E., Mu D., Goodson S., Powers S., Cordon-Cardo C., Lowe S.W., Hannon G.J. (2005). A microRNA polycistron as a potential human oncogene. Nature.

[4] Hayes J., Peruzzi P.P. (2014). MicroRNAs in cancer: Biomarkers, functions and therapy. Trends Mol. Med..

[5] Dews M., Homayouni A., Yu D., Murphy D., Sevignani C., Wentzel E., Furth E.E., Lee W.M., Enders G.H., Mendell J.T. (2006). Augmentation of tumor angiogenesis by a Myc-activated microRNA cluster. Nat. Genet..

[6] Motoyama K., Inoue H., Takatsuno Y., Tanaka F., Mimori K., Uetake H., Sugihara K., Mori M. (2009). Over- and under-expressed microRNAs in human colorectal cancer. Int. J. Oncol..

[7] Tsuchida A., Ohno S., Wu W., Borjigin N., Fujita K., Aoki T., Ueda S., Takanashi M., Kuroda M. (2011). miR-92 is a key oncogenic component of the miR-17-92 cluster in colon cancer. Cancer Sci..

[8] Yu G., Tang J.-Q., Tian M.-L., Li H., Wang X., Wu T., Zhu J., Huang S.-J., Wan Y.-L. (2012). Prognostic values of the miR-17-92 cluster and its paralogs in colon cancer. J. Surg. Oncol..

[9] Ng E.K., Chong W.W., Jin H., Lam E.K., Shin V.Y., Yu J., Poon T.C., Ng S.S., Sung J.J. (2009). Differential expression of microRNAs in plasma of patients with colorectal cancer: A potential marker for colorectal cancer screening. Gut.

[10] Reid J.F., Sokolova V., Zoni E., Lampis A., Pizzamiglio S., Bertan C., Zanutto S., Perrone F., Camerini T., Gallino G. (2012). miRNA profiling in colorectal cancer highlights miR-1 involvement in MET-dependent proliferation. Mol. Cancer Res. MCR.

[11] O’Brien J., Hayder H., Zayed Y., Peng C. (2018). Overview of MicroRNA Biogenesis, Mechanisms of Actions, and Circulation. Front. Endocrinol..

[12] Denli A.M., Tops B.B.J., Plasterk R.H.A., Ketting R.F., Hannon G.J. (2004). Processing of primary microRNAs by the Microprocessor complex. Nature.

[13] Ha M., Kim V.N. (2014). Regulation of microRNA biogenesis. Nat. Rev. Mol. Cell Biol..

[14] Yamamoto H., Adachi Y., Taniguchi H., Kunimoto H., Nosho K., Suzuki H., Shinomura Y. (2012). Interrelationship between microsatellite instability and microRNA in gastrointestinal cancer. World J. Gastroenterol..

[15] Al-Nakhle H., Burns P.A., Cummings M., Hanby A.M., Hughes T.A., Satheesha S., Shaaban A.M., Smith L., Speirs V. (2010). Estrogen receptor {beta}1 expression is regulated by miR-92 in breast cancer. Cancer Res..

[16] Wang C., Gao C., Zhuang J.-L., Ding C., Wang Y. (2012). A combined approach identifies three mRNAs that are down-regulated by microRNA-29b and promote invasion ability in the breast cancer cell line MCF-7. J. Cancer Res. Clin. Oncol..

[17] Svoronos A.A., Engelman D.M., Slack F.J. (2016). OncomiR or Tumor Suppressor? The Duplicity of MicroRNAs in Cancer. Cancer Res..

[18] Diosdado B., van de Wiel M.A., Terhaar Sive Droste J.S., Mongera S., Postma C., Meijerink W.J.H.J., Carvalho B., Meijer G.A. (2009). MiR-17-92 cluster is associated with 13q gain and c-myc expression during colorectal adenoma to adenocarcinoma progression. Br. J. Cancer.

[19] Mogilyansky E., Rigoutsos I. (2013). The miR-17/92 cluster: A comprehensive update on its genomics, genetics, functions and increasingly important and numerous roles in health and disease. Cell Death Differ..

[20] Smith A.L., Iwanaga R., Drasin D.J., Micalizzi D.S., Vartuli R.L., Tan A.-C., Ford H.L. (2012). The miR-106b-25 cluster targets Smad7, activates TGF-β signaling, and induces EMT and tumor initiating cell characteristics downstream of Six1 in human breast cancer. Oncogene.

[21] Li Y., Tan W., Neo T.W.L., Aung M.O., Wasser S., Lim S.G., Tan T.M.C. (2009). Role of the miR-106b-25 microRNA cluster in hepatocellular carcinoma. Cancer Sci..

[22] Zhou W., Shi G., Zhang Q., Wu Q., Li B., Zhang Z. (2014). MicroRNA-20b promotes cell growth of breast cancer cells partly via targeting phosphatase and tensin homologue (PTEN). Cell Biosci..

[23] Yamaguchi T., Iijima T., Wakaume R., Takahashi K., Matsumoto H., Nakano D., Nakayama Y., Mori T., Horiguchi S., Miyaki M. (2014). Underexpression of miR-126 and miR-20b in Hereditary and Nonhereditary Colorectal Tumors. Oncology.

[24] Sun Q., Zhang J., Cao W., Wang X., Xu Q., Yan M., Wu X., Chen W. (2013). Dysregulated miR-363 affects head and neck cancer invasion and metastasis by targeting podoplanin. Int. J. Biochem. Cell Biol..

[25] Zhou P., Huang G., Zhao Y., Zhong D., Xu Z., Zeng Y., Zhang Y., Li S., He F. (2014). MicroRNA-363-mediated downregulation of S1PR1 suppresses the proliferation of hepatocellular carcinoma cells. Cell. Signal..

[26] Ventura A., Young A.G., Winslow M.M., Lintault L., Meissner A., Erkeland S.J., Newman J., Bronson R.T., Crowley D., Stone J.R. (2008). Targeted Deletion Reveals Essential and Overlapping Functions of the miR-17-92 Family of miRNA Clusters. Cell.

[27] Martens-de Kemp S.R., Komor M.A., Hegi R., Bolijn A.S., Tijssen M., de Groen F.L., Depla A., van Leerdam M., Meijer G.A., Fijneman R.J. (2022). Overexpression of the miR-17-92 cluster in colorectal adenoma organoids causes a carcinoma-like gene expression signature. Neoplasia.

[28] Zhang G.-J., Li L.-F., Yang G.-D., Xia S.-S., Wang R., Leng Z.-W., Liu Z.-L., Tian H.-P., He Y., Meng C.-Y. (2017). MiR-92a promotes stem cell-like properties by activating Wnt/β-catenin signaling in colorectal cancer. Oncotarget.

[29] Huang Z., Yang M. (2022). Molecular Network of Colorectal Cancer and Current Therapeutic Options. Front. Oncol..

[30] Disoma C., Zhou Y., Li S., Peng J., Xia Z. (2022). Wnt/β-catenin signaling in colorectal cancer: Is therapeutic targeting even possible?. Biochimie.

[31] Li Y., Lauriola M., Kim D., Francesconi M., D’Uva G., Shibata D., Malafa M.P., Yeatman T.J., Coppola D., Solmi R. (2016). Adenomatous polyposis coli (APC) regulates miR17-92 cluster through β-catenin pathway in colorectal cancer. Oncogene.

[32] Jiang H., Wang P., Wang Q., Wang B., Mu J., Zhuang X., Zhang L., Yan J., Miller D., Zhang H.-G. (2014). Quantitatively Controlling Expression of miR-17-92 Determines Colon Tumor Progression in a Mouse Tumor Model. Am. J. Pathol..

[33] Yuan G., Liu B., Han W., Zhao D. (2019). LncRNA-MIR17HG mediated upregulation of miR-17 and miR-18a promotes colon cancer progression via activating Wnt/β-catenin signaling. Transl. Cancer Res..

[34] Ma Y., Zhang P., Wang F., Zhang H., Yang Y., Shi C., Xia Y., Peng J., Liu W., Yang Z. (2012). Elevated oncofoetal miR-17-5p expression regulates colorectal cancer progression by repressing its target gene P130. Nat. Commun..

[35] Yu F.-B., Sheng J., Yu J.-M., Liu J.-H., Qin X.-X., Mou B. (2020). MiR-19a-3p regulates the Forkhead box F2-mediated Wnt/β-catenin signaling pathway and affects the biological functions of colorectal cancer cells. World J. Gastroenterol..

[36] Song K., Liu C., Zhang J., Yao Y., Xiao H., Yuan R., Li K., Yang J., Zhao W., Zhang Y. (2022). Integrated multi-omics analysis reveals miR-20a as a regulator for metabolic colorectal cancer. Heliyon.

[37] Sun T., Yin Y.-F., Jin H.-G., Liu H.-R., Tian W.-C. (2022). Exosomal microRNA-19b targets FBXW7 to promote colorectal cancer stem cell stemness and induce resistance to radiotherapy. Kaohsiung J. Med. Sci..

[38] Wee P., Wang Z. (2017). Epidermal Growth Factor Receptor Cell Proliferation Signaling Pathways. Cancers.

[39] Pabla B., Bissonnette M., Konda V.J. (2015). Colon cancer and the epidermal growth factor receptor: Current treatment paradigms, the importance of diet, and the role of chemoprevention. World J. Clin. Oncol..

[40] Wei S., Hu W., Feng J., Geng Y. (2022). Promotion or remission: A role of noncoding RNAs in colorectal cancer resistance to anti-EGFR therapy. Cell Commun. Signal. CCS.

[41] Velho S., Oliveira C., Ferreira A., Ferreira A.C., Suriano G., Schwartz S.J., Duval A., Carneiro F., Machado J.C., Hamelin R. (2005). The prevalence of PIK3CA mutations in gastric and colon cancer. Eur. J. Cancer.

[42] Fang L., Li H., Wang L., Hu J., Jin T., Wang J., Yang B.B. (2014). MicroRNA-17-5p promotes chemotherapeutic drug resistance and tumour metastasis of colorectal cancer by repressing PTEN expression. Oncotarget.

[43] Michas A., Michas V., Anagnostou E., Galanopoulos M., Tolia M., Tsoukalas N. (2023). The Clinical Significance of MicroRNAs in Colorectal Cancer Signaling Pathways: A Review. Glob. Med. Genet..

[44] Cellura D., Pickard K., Quaratino S., Parker H., Strefford J.C., Thomas G.J., Mitter R., Mirnezami A.H., Peake N.J. (2015). miR-19-Mediated Inhibition of Transglutaminase-2 Leads to Enhanced Invasion and Metastasis in Colorectal Cancer. Mol. Cancer Res. MCR.

[45] Principe D.R., Doll J.A., Bauer J., Jung B., Munshi H.G., Bartholin L., Pasche B., Lee C., Grippo P.J. (2014). TGF-β: Duality of function between tumor prevention and carcinogenesis. J. Natl. Cancer Inst..

[46] Lin E., Kuo P.-H., Liu Y.-L., Yang A.C., Tsai S.-J. (2017). Transforming growth factor-β signaling pathway-associated genes SMAD2 and TGFBR2 are implicated in metabolic syndrome in a Taiwanese population. Sci. Rep..

[47] Cheng D., Zhao S., Tang H., Zhang D., Sun H., Yu F., Jiang W., Yue B., Wang J., Zhang M. (2016). MicroRNA-20a-5p promotes colorectal cancer invasion and metastasis by downregulating Smad4. Oncotarget.

[48] Lv H., Zhang Z., Wang Y., Li C., Gong W., Wang X. (2016). MicroRNA-92a Promotes Colorectal Cancer Cell Growth and Migration by Inhibiting KLF4. Oncol. Res..

[49] Nishida N., Yano H., Nishida T., Kamura T., Kojiro M. (2006). Angiogenesis in cancer. Vasc. Health Risk Manag..

[50] Ma H., Pan J.-S., Jin L.-X., Wu J., Ren Y.-D., Chen P., Xiao C., Han J. (2016). MicroRNA-17~92 inhibits colorectal cancer progression by targeting angiogenesis. Cancer Lett..

[51] Yamada N.O., Heishima K., Akao Y., Senda T. (2019). Extracellular Vesicles Containing MicroRNA-92a-3p Facilitate Partial Endothelial-Mesenchymal Transition and Angiogenesis in Endothelial Cells. Int. J. Mol. Sci..

[52] Lorsy E., Topuz A.S., Geisler C., Stahl S., Garczyk S., von Stillfried S., Hoss M., Gluz O., Hartmann A., Knüchel R. (2016). Loss of Dickkopf 3 Promotes the Tumorigenesis of Basal Breast Cancer. PLoS ONE.

[53] Al Shareef Z., Kardooni H., Murillo-Garzón V., Domenici G., Stylianakis E., Steel J.H., Rabano M., Gorroño-Etxebarria I., Zabalza I., Vivanco M.d.M. (2018). Protective effect of stromal Dickkopf-3 in prostate cancer: Opposing roles for TGFBI and ECM-1. Oncogene.

[54] Guo Q., Qin W. (2015). DKK3 blocked translocation of β-catenin/EMT induced by hypoxia and improved gemcitabine therapeutic effect in pancreatic cancer Bxpc-3 cell. J. Cell. Mol. Med..

[55] Zhou L., Husted H., Moore T., Lu M., Deng D., Liu Y., Ramachandran V., Arumugam T., Niehrs C., Wang H. (2018). Suppression of stromal-derived Dickkopf-3 (DKK3) inhibits tumor progression and prolongs survival in pancreatic ductal adenocarcinoma. Sci. Transl. Med..

[56] Busceti C.L., Marchitti S., Bianchi F., Di Pietro P., Riozzi B., Stanzione R., Cannella M., Battaglia G., Bruno V., Volpe M. (2017). Dickkopf-3 Upregulates VEGF in Cultured Human Endothelial Cells by Activating Activin Receptor-Like Kinase 1 (ALK1) Pathway. Front. Pharmacol..

[57] Zitt M., Untergasser G., Amberger A., Moser P., Stadlmann S., Zitt M., Müller H.M., Mühlmann G., Perathoner A., Margreiter R. (2008). Dickkopf-3 as a new potential marker for neoangiogenesis in colorectal cancer: Expression in cancer tissue and adjacent non-cancerous tissue. Dis. Markers.

[58] Safari E., Mosayebi G., Khorram S. (2018). Dkk-3 as a potential biomarker for diagnosis and prognosis of colorectal cancer. Med. J. Islam. Repub. Iran.

[59] Wei Q.-D., Zheng W.-B., Sun K., Xue Q., Yang C.-Z., Li G.-X. (2019). MiR-92a promotes the invasion and migration of colorectal cancer by targeting RECK. Int. J. Clin. Exp. Pathol..

[60] Pidíková P., Herichová I. (2021). miRNA Clusters with Up-Regulated Expression in Colorectal Cancer. Cancers.

[61] Jepsen R.K., Novotny G.W., Klarskov L.L., Bang-Berthelsen C.H., Haakansson I.T., Hansen A., Christensen I.J., Riis L.B., Høgdall E. (2018). Early metastatic colorectal cancers show increased tissue expression of miR-17/92 cluster members in the invasive tumor front. Hum. Pathol..

[62] Fu F., Jiang W., Zhou L., Chen Z. (2018). Circulating Exosomal miR-17-5p and miR-92a-3p Predict Pathologic Stage and Grade of Colorectal Cancer. Transl. Oncol..

[63] Zhou T., Zhang G., Liu Z., Xia S., Tian H. (2013). Overexpression of miR-92a correlates with tumor metastasis and poor prognosis in patients with colorectal cancer. Int. J. Color. Dis..

[64] Zhang G., Li Y., Zhou H., Xiao H., Zhou T. (2014). miR-20a is an independent prognostic factor in colorectal cancer and is involved in cell metastasis. Mol. Med. Rep..

[65] Lai H., Zhang J., Zuo H., Liu H., Xu J., Feng Y., Lin Y., Mo X. (2020). Overexpression of miR-17 is correlated with liver metastasis in colorectal cancer. Medicine.

[66] Ke T.-W., Wei P.-L., Yeh K.-T., Chen W.T.-L., Cheng Y.-W. (2015). MiR-92a Promotes Cell Metastasis of Colorectal Cancer Through PTEN-Mediated PI3K/AKT Pathway. Ann. Surg. Oncol..

[67] Kim T.W., Lee Y.S., Yun N.H., Shin C.H., Hong H.K., Kim H.H., Cho Y.B. (2020). MicroRNA-17-5p regulates EMT by targeting vimentin in colorectal cancer. Br. J. Cancer.

[68] Hu J.L., Wang W., Lan X.L., Zeng Z.C., Liang Y.S., Yan Y.R., Song F.Y., Wang F.F., Zhu X.H., Liao W.J. (2019). CAFs secreted exosomes promote metastasis and chemotherapy resistance by enhancing cell stemness and epithelial-mesenchymal transition in colorectal cancer. Mol. Cancer.

[69] Sun K., Chen L., Li Y., Huang B., Yan Q., Wu C., Lai Q., Fang Y., Cai J., Liu Y. (2023). METTL14-dependent maturation of pri-miR-17 regulates mitochondrial homeostasis and induces chemoresistance in colorectal cancer. Cell Death Dis..

[70] Zhao H.-Y., Ooyama A., Yamamoto M., Ikeda R., Haraguchi M., Tabata S., Furukawa T., Che X.-F., Iwashita K., Oka T. (2008). Down regulation of c-Myc and induction of an angiogenesis inhibitor, thrombospondin-1, by 5-FU in human colon cancer KM12C cells. Cancer Lett..

[71] Badr D., Fouad M.A., Hussein M., Salem S., Zekri A., Shouman S. (2023). Rebound increase in microRNA levels at the end of 5-FU-based therapy in colorectal cancer patients. Sci. Rep..

[72] Khoury S., Tran N. (2015). Circulating microRNAs: Potential biomarkers for common malignancies. Biomark. Med..

[73] Treiber T., Treiber N., Meister G. (2019). Regulation of microRNA biogenesis and its crosstalk with other cellular pathways. Nat. Rev. Mol. Cell Biol..

[74] Carter J.V., Galbraith N.J., Yang D., Burton J.F., Walker S.P., Galandiuk S. (2017). Blood-based microRNAs as biomarkers for the diagnosis of colorectal cancer: A systematic review and meta-analysis. Br. J. Cancer.

[75] Toiyama Y., Okugawa Y., Fleshman J., Boland C.R., Goel A. (2018). MicroRNAs as Potential Liquid Biopsy Biomarkers in Colorectal Cancer: A Systematic Review. Biochim. Biophys. Acta Rev. Cancer.

[76] de Miguel Pérez D., Rodriguez Martínez A., Ortigosa Palomo A., Delgado Ureña M., Garcia Puche J.L., Robles Remacho A., Exposito Hernandez J., Lorente Acosta J.A., Ortega Sánchez F.G., Serrano M.J. (2020). Extracellular vesicle-miRNAs as liquid biopsy biomarkers for disease identification and prognosis in metastatic colorectal cancer patients. Sci. Rep..

[77] Han J., Sun W., Liu R., Zhou Z., Zhang H., Chen X., Ba Y. (2020). Plasma Exosomal miRNA Expression Profile as Oxaliplatin-Based Chemoresistant Biomarkers in Colorectal Adenocarcinoma. Front. Oncol..

[78] Huang Z., Huang D., Ni S., Peng Z., Sheng W., Du X. (2010). Plasma microRNAs are promising novel biomarkers for early detection of colorectal cancer. Int. J. Cancer.

[79] Liu G.-H., Zhou Z.-G., Chen R., Wang M.-J., Zhou B., Li Y., Sun X.-F. (2013). Serum miR-21 and miR-92a as biomarkers in the diagnosis and prognosis of colorectal cancer. Tumour Biol. J. Int. Soc. Oncodevelopmental Biol. Med..

[80] Wu C.W., Ng S.S.M., Dong Y.J., Ng S.C., Leung W.W., Lee C.W., Wong Y.N., Chan F.K.L., Yu J., Sung J.J.Y. (2012). Detection of miR-92a and miR-21 in stool samples as potential screening biomarkers for colorectal cancer and polyps. Gut.

[81] Yau T.O., Wu C.W., Dong Y., Tang C.-M., Ng S.S.M., Chan F.K.L., Sung J.J.Y., Yu J. (2014). microRNA-221 and microRNA-18a identification in stool as potential biomarkers for the non-invasive diagnosis of colorectal carcinoma. Br. J. Cancer.

[82] Yau T.O., Wu C.W., Tang C.-M., Chen Y., Fang J., Dong Y., Liang Q., Ng S.S.M., Chan F.K.L., Sung J.J.Y. (2016). MicroRNA-20a in human faeces as a non-invasive biomarker for colorectal cancer. Oncotarget.

[83] Chang P.-Y., Chen C.-C., Chang Y.-S., Tsai W.-S., You J.-F., Lin G.-P., Chen T.-W., Chen J.-S., Chan E.-C. (2016). MicroRNA-223 and microRNA-92a in stool and plasma samples act as complementary biomarkers to increase colorectal cancer detection. Oncotarget.

[84] Koga Y., Yasunaga M., Takahashi A., Kuroda J., Moriya Y., Akasu T., Fujita S., Yamamoto S., Baba H., Matsumura Y. (2010). MicroRNA expression profiling of exfoliated colonocytes isolated from feces for colorectal cancer screening. Cancer Prev. Res..

[85] Choi H.H., Cho Y.-S., Choi J.H., Kim H.-K., Kim S.S., Chae H.-S. (2019). Stool-Based miR-92a and miR-144* as Noninvasive Biomarkers for Colorectal Cancer Screening. Oncology.

[86] Rotelli M.T., Di Lena M., Cavallini A., Lippolis C., Bonfrate L., Chetta N., Portincasa P., Altomare D.F. (2015). Fecal microRNA profile in patients with colorectal carcinoma before and after curative surgery. Int. J. Color. Dis..

[87] Viswanathan V., Opdenaker L., Modarai S., Fields J.Z., Gonye G., Boman B.M. (2020). MicroRNA Expression Profiling of Normal and Malignant Human Colonic Stem Cells Identifies miRNA92a as a Regulator of the LRIG1 Stem Cell Gene. Int. J. Mol. Sci..

